# Combined transcriptomic and metabolomic analyses uncover rearranged gene expression and metabolite metabolism in tobacco during cold acclimation

**DOI:** 10.1038/s41598-020-62111-x

**Published:** 2020-03-23

**Authors:** Jiayang Xu, Zheng Chen, Fazhan Wang, Wei Jia, Zicheng Xu

**Affiliations:** National Tobacco Cultivation and Physiology and Biochemistry Research Center, College of Tobacco Science, Henan Agricultural University, Zhengzhou, 450002 People’s Republic of China

**Keywords:** Biological techniques, Plant sciences

## Abstract

Cold temperatures often severely restrict the growth, distribution and productivity of plants. The freezing tolerance of plants from temperate climates can be improved by undergoing periods of cold acclimation (CA). Tobacco is an important economic plant and is sensitive to cold stress. However, the dynamic changes and regulatory mechanisms of gene expression and metabolic processes during CA remain largely unknown. In this study, we performed RNA sequencing and metabolomic profiling analyses to identify the genes and metabolites specifically expressed during CA. Our transcriptomic data revealed 6905 differentially expressed genes (DEGs) during CA. Functional annotation and enrichment analyses revealed that the DEGs were involved mainly in signal transduction, carbohydrate metabolism and phenylpropanoid biosynthesis. Moreover, a total of 35 significantly changed metabolites were identified during CA via an LC-MS platform. Many protective metabolites, such as amino acids, carbohydrates, tricarboxylic acid (TCA) cycle intermediates and phenylpropanoid-related substances, were identified during CA. The gene-metabolite network extensively outlined the biological processes associated with the utilization of sugars, activation of amino acid metabolism, TCA cycle and phenylpropanoid biosynthesis in tobacco under CA. The results of our present study provide a comprehensive view of signal transduction and regulation, gene expression and dynamic changes in metabolites during CA.

## Introduction

Land plants living in restricted spaces usually encounter environmental stresses that affect their growth and productivity. Among these stressful conditions, cold is one of the major constraining factors that limits the geographical distribution and agricultural productivity of crops^1^. Tobacco (*Nicotiana tabacum* L.), a *Solanaceae* species, originates from tropical and subtropical America but is now commercially cultivated worldwide due to its high economic value. In addition, it is commonly used as an excellent model plant species for studies on physiological traits and molecular functions. However, tobacco is sensitive to cold stress^2^. Cold acclimation (CA) is recognized as a typical process by which many temperate plants develop chilling and freezing tolerance^3^. Hence, exploring the molecular mechanism of CA in tobacco is of great value for breeding cold-tolerant varieties for agricultural production.

Numerous studies have shown that CA is closely associated with complex changes in the regulation of gene expression, accumulation of protective metabolites and maintenance of metabolic homeostasis^4–6^. Many efforts in CA studies of various plant species have suggested that cold induces extensive recombination of gene transcription^7–9^. At present, the product of the best-known cold-responsive (COR) gene involved in the CA signalling pathway is the ICE1-CBF-COR module. In this model, *Arabidopsis* plants exposed to cold stress induce the expression of C-repeat (CRT) binding factors/dehydration-responsive element binding factors (CBFs/DREBs), which can bind to the promoter of COR genes to regulate their transcription^10,11^. Despite these valuable insights, the whole understanding of plants during CA in terms of the potential links between gene expression and metabolite regulatory networks remains largely unknown.

Modern metabolomics techniques enable the monitoring of low-molecular-weight metabolites to gain a comprehensive understanding of biological processes during exposure to various stresses^12,13^. The identification of general cold responses has generated metabonomic data from plants growing during CA. For example, the accumulation of sugars and the synthesis of amino acids are well recognized as essential strategies during CA not only because both sugars and amino acids are signalling molecules but also because they are precursors of large amounts of metabolites with multiple stress functions^10,14^. Recent technological advances from the combination of transcriptomic and metabolomic analyses have provided several possible approaches for dissecting molecular features involved in the re-adjustment of gene expression and metabolic pathways. Using an integrated genetic and metabolite analyses, Jia *et al*. detected several genes and metabolites that were specifically modulated in response to various stages of drought stress in *Astragalus membranaceus*^15^. Moreover, Moschen *et al*. integrated transcriptomic and metabolic data to reveal alterations in the levels of hub transcription factors (TFs) and metabolites involved in the regulation of drought stress tolerance in sunflower^16^.

Therefore, the objectives of this work were to investigate, on a large scale, the transcriptomic and metabolomic changes in cold-acclimated tobacco plants. Moreover, we uncovered a potential regulatory network between genes and metabolites that are crucial during CA. The outcomes of the present study provide a comprehensive framework for better understanding the potential molecular adaptation strategy of tobacco in response to cold stress at the transcriptomic and metabolomic levels.

## Results

### Physiological changes in tobacco plants during cold stress

**Figure 1:**
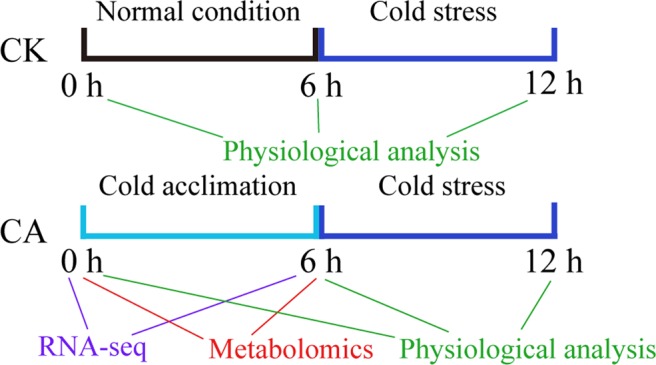
Schematic flowchart of the experimental settings and research strategy applied in this study. For the control treatment (CK), tobacco plants were exposed to cold stress for 6 h; for cold acclimation (CA) treatment, tobacco plants were first transferred to 15 °C conditions for 6 h and then exposed to cold stress. Physiological data were collected at 0 h, 6 h and 12 h, and RNA-seq and metabolomics data were collected after 0 h and 6 h of CA treatment.

We first measured the physiological and biochemical changes in tobacco plants during different phases of a moderate low temperature (15 °C) at 6 h, 12 h and 24 h. As shown in Fig. S1, during the whole treatment, moderate low temperature had no impact on the dry weight (DW), whereas low temperature for 24 h significantly reduced the fresh weight (FW). Moderate low temperature also markedly induced electrolyte leakage (EL) and increased the malondialdehyde (MDA) content at both 12 h and 24 h. Considering the growth conditions of tobacco seedlings, 6 h of 15 °C treatment was selected as our low temperature pre-treatment. Our further experimental design is shown in Fig. 1. For cold-acclimated tobacco plants, seedlings at one of five stages were first transferred to 15 °C conditions for 6 h. Afterward, both the control (CK; without CA pre-treatment) and cold-acclimated tobacco plants were exposed to cold stress (4 °C) for 6 h.

**Figure 2:**
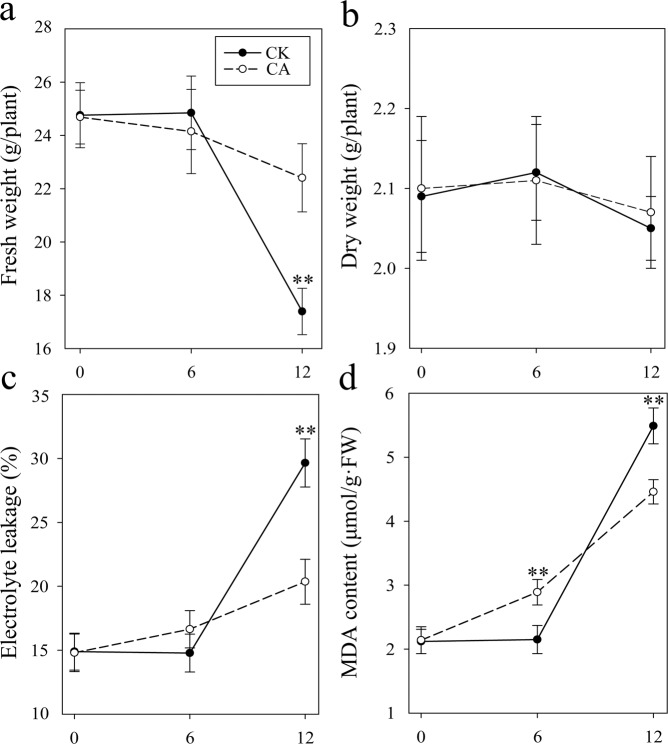
Physiological changes in tobacco plants under low temperature. Effect of cold acclimation (CA; 6 h) and cold stress (12 h) on the fresh weight (**a**), dry weight (**b**), electrolyte leakage (**c**) and MDA content (**d**) of tobacco seedlings. Values are the means ± SDs (n = 5). **Indicates a significant difference at *p* < 0.05 evaluated by the LSD test between the CK and CA treatments at the same sampling point.

The effects of CA on tobacco plants is shown in Fig. 2. Cold stress caused dramatic, obvious damage to tobacco seedlings, resulting in reduced FW and increased levels of EL and MDA. However, compared with the CK samples, the cold-acclimated tobacco plants presented 28.87% greater FW and 31.33%, 18.76% lower EL and MDA content, respectively, indicating that CA relieved the damage induced by cold stress in tobacco. Notably, there were no marked changes in DW throughout the whole test; this finding may highlight the importance of molecular changes during CA treatment.

### RNA sequencing results of the cold-acclimated tobacco plants

**Figure 3:**
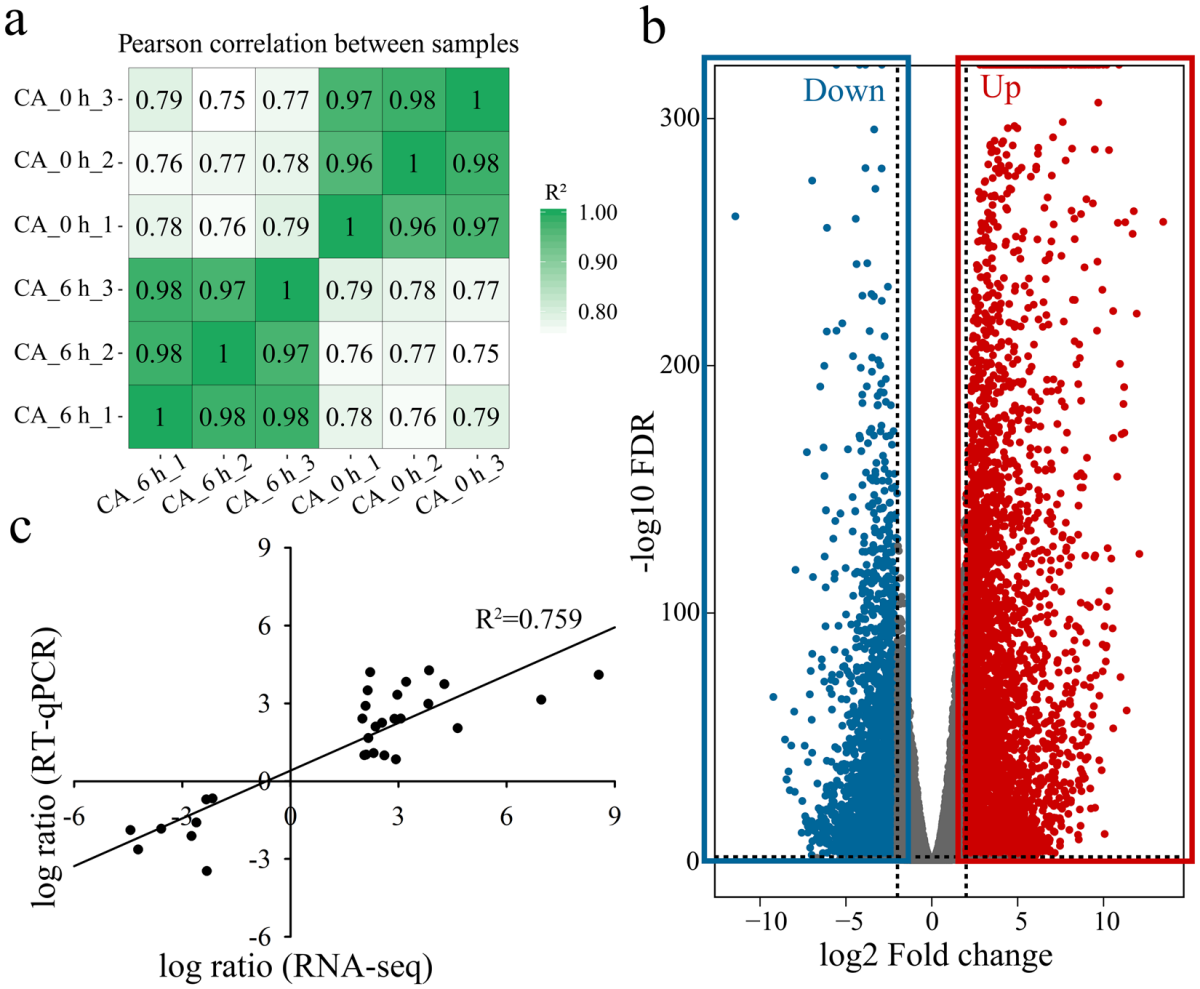
General analysis of transcriptome sequences in cold-acclimated tobacco plants. Heatmap of Pearson correlations of the expression levels among samples (**a**). Volcano plot of differentially expressed genes (DEGs) between CA_6 h and CA_0 h treatment (**b**). Validation of transcriptomic data (30 DEGs) by RT-qPCR (**c**).

To explore the cold-regulated transcriptome of tobacco during CA, the total mRNA from the cold-acclimated samples (0 h and 6 h) was sequenced using the Illumina platform, with three biological replicates per treatment. A total of approximately 314 million paired-end reads were generated from six libraries. After trimming the low-quality reads from the raw data, we obtained 27.29 Gb and 19.10 Gb of clean reads in the CA_0 h and CA_6 h groups, respectively (Table 1). Pearson correlation analysis was conducted to determine the gene expression reliability among the samples. High reproducibility between biological replicates was observed (R^2^ > 0.96) for all treatments (Fig. 3a).

**Table 1:** Summary of the RNA sequencing datasets.

Treatment	Raw Reads	Clean Reads	Clean Bases	Q30 (%)	Mapped Reads
CA_0 h_1	61720312	60676746	9.10 G	89.88	56771771 (93.56%)
CA_0 h_2	66990430	66124118	9.92 G	88.92	61394014 (92.85%)
CA_0 h_3	55908862	55117224	8.27 G	89.99	51586655 (93.59%)
CA_6 h_1	47377736	46723626	7.01 G	87.23	42091209 (90.09%)
CA_6 h_2	41048714	40093972	6.01 G	90.37	37205119 (92.79%)
CA_6 h_3	41172760	40535282	6.08 G	89.60	37283555 (91.98%)

To investigate the pivotal genes in tobacco under CA treatment, differentially expressed genes (DEGs) were identified according to the strict criteria of |log_2_ fold change (FC) | > 2 and a false discovery rate-adjusted *p* value (FDR) < 0.05. A total of 6905 genes, of which the expression of 3421 and 3484 was up- and downregulated, respectively, were found to be differentially expressed during CA (Fig. 3b). To validate the mRNA sequencing data, thirty DEGs that were determined to be important for CA (they are involved mainly in cold signal transduction, carbohydrate and phenylpropanoid biosynthesis metabolism) were manually selected and analysed by RT-qPCR (Fig. 3c, Table S1). As shown in Fig. 3c, the trends of the fragments per kilobase of the transcripts per million fragments mapped (FPKM) in the transcriptomic data and the relative FC of gene expression from the RT-qPCR data were the same, showing the reliability of RNA-seq.

**Figure 4:**
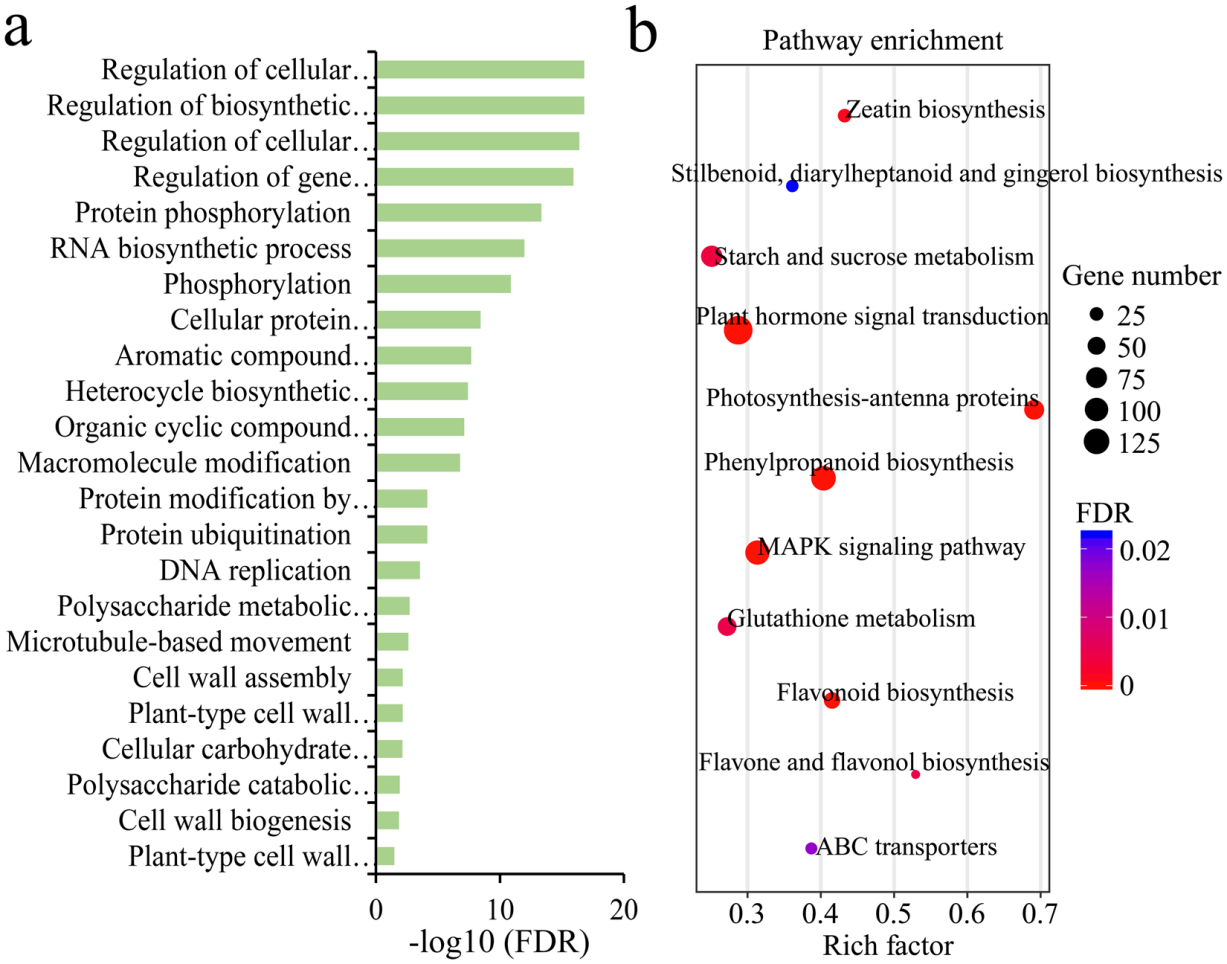
Functional annotation and enrichment analysis of differentially expressed genes (DEGs) under CA. GO enrichment of the DEGs in biological progress (**a**). KEGG pathway analysis of DEGs (**b**).

Gene Ontology (GO) and Kyoto Encyclopedia of Genes and Genomes (KEGG) pathway enrichment analyses were performed to explore the functions of DEGs under CA. The 6905 DEGs were significantly enriched in 64 GO terms with respect to the biological process, cellular component and molecular function categories (Table S2). Among the GO terms, DEGs enriched in biological progress were related to “regulation of biosynthetic process”, “protein phosphorylation”, “cellular protein modification process”, “protein ubiquitination” and “cell wall assembly” (Fig. 4a). The KEGG analysis resulted in four significant KEGG pathways for the biosynthesis of secondary metabolites (e.g., “phenylpropanoid biosynthesis”, “flavonoid biosynthesis”), two pathways for signal transduction (e.g., “MAPK signaling pathway” and “plant hormone signal transduction”) and one pathway for carbohydrate metabolism (e.g., “starch and sucrose metabolism”) (Fig. 4b, Table S3).

### Metabolic analysis of cold-acclimated tobacco

**Figure 5:**
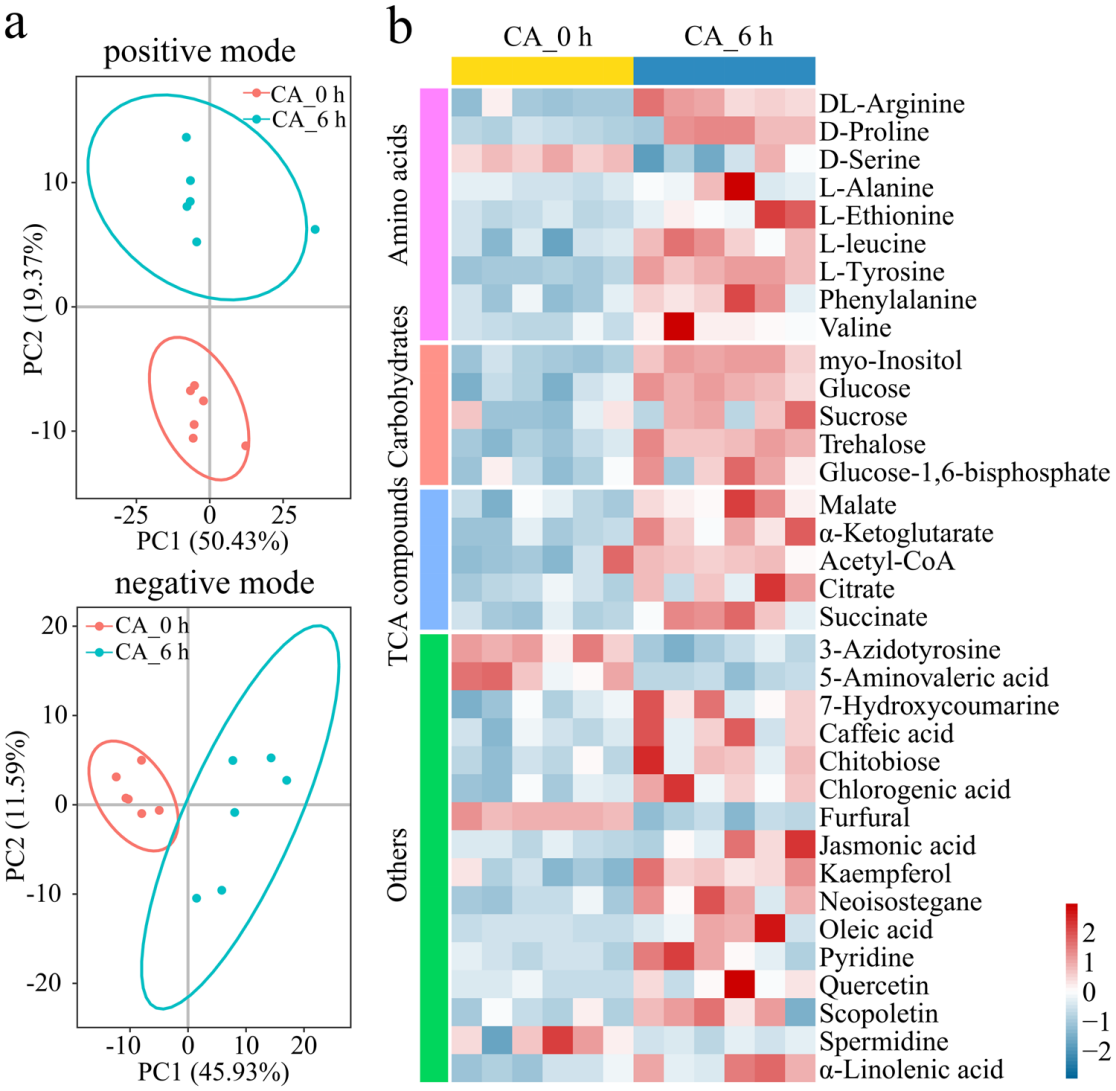
Metabolite changes from CA treatment. Score plot of the principal component analysis (PCA) based on LC/MS data (**a**). Heatmap of the differentially accumulated metabolites (relative abundance) between the CA_6 h and CA_0 h samples (**b**).

The metabolic profiles of the tobacco leaves of the cold-acclimated plant samples were investigated via LC-MS. A total of 116 and 79 unique metabolites were detected in the positive mode and negative mode, respectively (Table S4). Principal component analysis (PCA) was conducted to visualize the differences among the treatments. The score plot showed clear separation in both the positive mode and negative mode (Fig. 5a), indicating a significant change in the metabolite profiles during the course of CA.

Orthogonal partial least squares discriminant analysis (OPLS-DA) was performed to identify the metabolites that significantly changed. On the basis of a variable importance in projection (VIP) score >1, an FC > 1.5 or <0.66 and *p* < 0.05, a total of 35 metabolites that significantly varied during CA were identified in positive mode and negative mode (Fig. 5b, Table S5). As shown in Fig. 5b, the majority of the amino acids markedly accumulated when the plants were subjected to CA, showing the potential roles of these compounds in protein metabolism and the stress response during CA. Moreover, CA also triggered an increase in the levels of sugar metabolism and tricarboxylic acid (TCA) cycle-related metabolites, including a 1.99-fold increase in myo-inositol, a 2.21-fold increase in glucose, a 1.52-fold increase in sucrose, a 3.34-fold increase in trehalose, a 1.86-fold increase in glucose-1,6-bisphosphate, a 1.53-fold increase in malate, a 2.77-fold increase in α-ketoglutarate, a 1.58-fold increase in acetyl-CoA, a 1.83-fold increase in citrate and a 1.60-fold increase in succinate. These results indicated that CA induced dramatic changes in substances and energy metabolism by the massive reprogramming of metabolome.

### Integrated analysis of the transcriptome and metabolome

**Figure 6:**
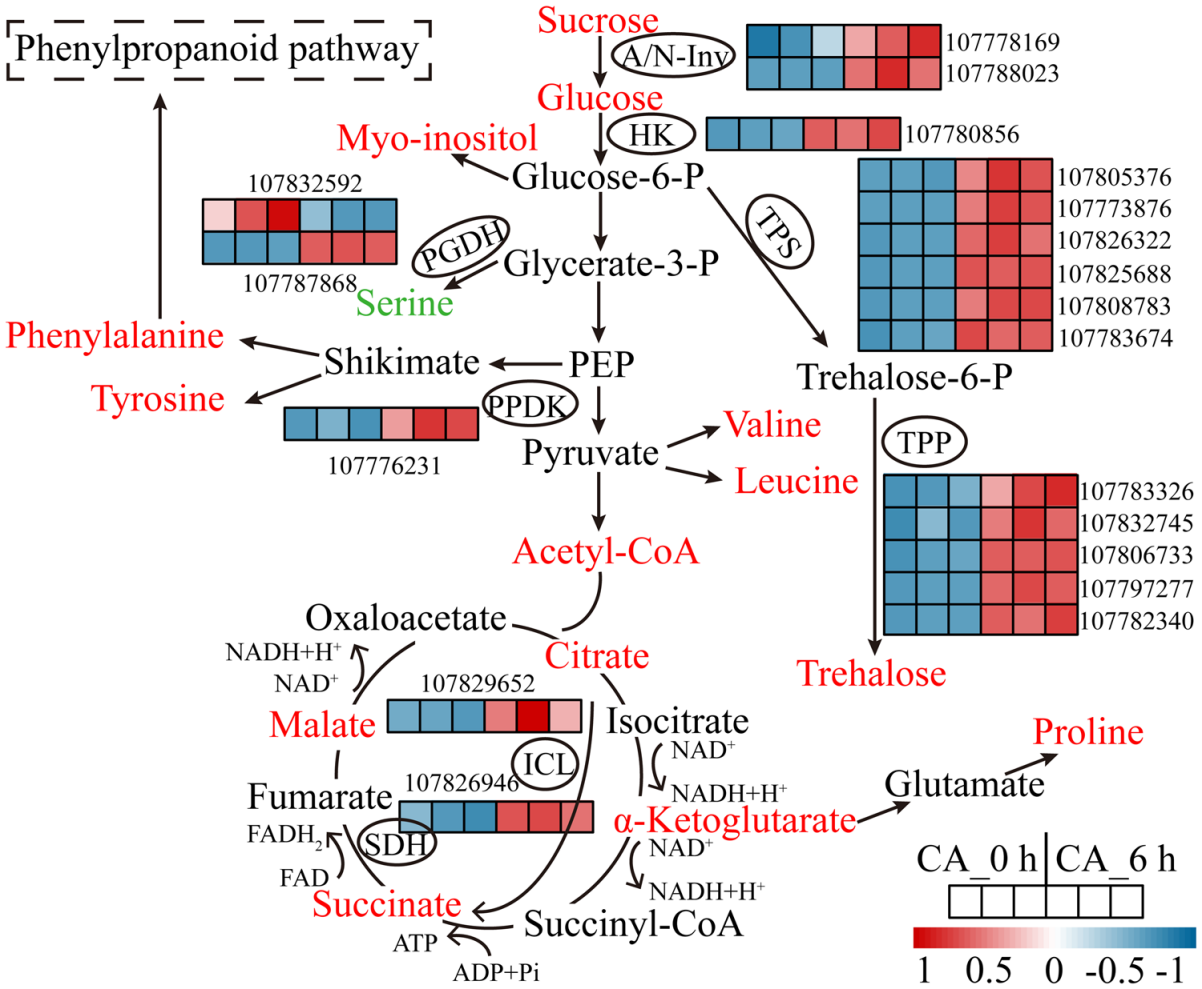
Map of differentially expressed genes (DEGs) and metabolites involved in carbohydrate, amino acid and TCA metabolite pathways. The metabolites in red and green represent upregulated and downregulated accumulation under CA, respectively. The genes with ellipses were differentially expressed, and their relative expression levels (FPKM value) in the CA_0 h and CA_6 h samples (three biological replicates of each treatment) are shown as heatmaps. Abbreviations: A/N-Inv, neutral/alkaline invertase; HK, hexokinase; TPS, trehalose-phosphate synthase; TPP, trehalose-phosphate phosphatase; PGDH, D-3-phosphoglycerate dehydrogenase; PPDK, pyruvate phosphate dikinase; SDH, succinate dehydrogenase; ICL, isocitrate lyase.

Notably, DEGs were markedly enriched in “polysaccharide metabolic process” terms and “starch and sucrose metabolism” pathway, suggesting that sugars act as important signalling molecules and provide an energy source for tobacco plants during CA. To obtain a more comprehensive understanding of the regulatory mechanism during CA, we combined our RNA-seq and metabolomic data to construct a gene-metabolite network. As shown in Fig. 6, both the transcripts and metabolites involved in the metabolism of carbohydrates (e.g., *NtA/N-Inv*, *NtHK*, *NtTPS*, *NtTPP*, sucrose, glucose, myo-inositol, trehalose), amino acids (e.g., *NtPGDH*, serine, tyrosine, phenylalanine, proline) and the TCA cycle (e.g., *NtICL*, *NtSDH*, citrate, α-ketoglutarate, succinate, malate) were significantly regulated under CA, indicating their potential roles in adjustments to CA-mediated homeostasis and coping with subsequent cold stress.

**Figure 7:**
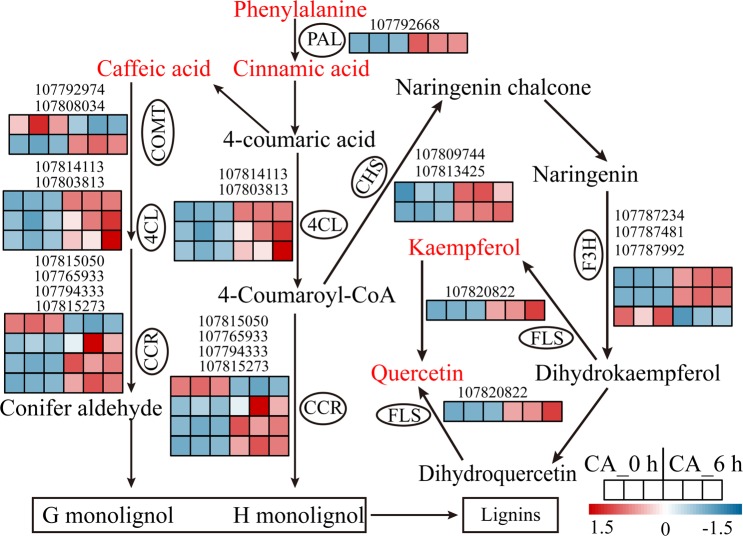
Map of differentially expressed genes (DEGs) and metabolites involved in the phenylpropanoid biosynthesis pathway. The image was modified from map00940 (version 5/14/19) from the KEGG database (http://www.kegg.jp). The metabolites in red represent upregulated accumulation under CA. The genes with ellipses were differentially expressed, and their relative expression levels (FPKM value) in the CA_0 h and CA_6 h samples (three biological replicates of each treatment) are shown as heatmaps. Abbreviations: PAL, phenylalanine ammonia lyase; 4CL, 4-coumarate-CoA ligase; CHS, chalcone synthase; COMT, caffeic acid-O-methyltransferase; CCR, cinnamoyl-CoA reductase; FLS, flavonol synthase; F3H, flavanone 3-hydroxylase.

Cold also significantly affects phenylpropanoid biosynthesis, resulting in the accumulation of lignin and flavonoids^17,18^. Our combined transcriptomic data and metabolite profiling revealed that most of the DEGs and metabolites in the phenylpropanoid biosynthesis process were upregulated (Fig. 7). For example, *PAL* catalyses the deamination of phenylalanine to cinnamic acid. In addition, *COMT*, *4CL* and *CCR* catalyse the conversion of caffeic acid into conifer aldehyde to form G monolignol. Kaempferol and quercetin, two major flavonoids, also increased in abundance during CA. The increased levels of gene expression and metabolites that participate in the phenylpropanoid biosynthesis pathway suggest that the regulation of lignin and flavonoids plays an essential role in the plant response to low temperature.

## Discussion

The ability of plants to generate and utilize energy is a basic adjustment during CA^19^. Energy metabolites such as sugars and ATP are considered the most important factors in cold tolerance^20,21^. Sucrose is a key molecule in cellular biosynthesis and can be cleaved to glucose and fructose to provide energy^22^. Neutral/alkaline invertase (A/N-Inv) is one of the invertases that catalyses the irreversible hydrolysis of sucrose. A previous study showed that cold could induce the expression of A/N-Inv genes^23^. Here, the higher levels of sucrose and glucose content and A/N-Inv gene expression may help promote the function of mitochondria and produce additional ATP under cold conditions^24^. Furthermore, trehalose is an important compatible solute in plants that can function as an osmolyte to stabilize membranes and is implicated in responses to cold and salinity^25,26^. Here, the members of two families of genes encoding trehalose-phosphate synthase (TPS) and trehalose-phosphate phosphatase (TPP), which catalyse the synthesis of trehalose via the intermediate trehalose-6-phosphate in a two-step process, were upregulated in cold treatment (Fig. 6, Table S6). It has been demonstrated that overexpression of TPS and TPP can promote plant abiotic stress tolerance by activating trehalose metabolism^14^. In addition to these increased accumulations of sugar metabolites, the expression levels of many energy transfer-related proteins, such as ADP/ATP carrier proteins, calcium-transporting ATPase and cadmium/zinc-transporting ATPase, also increased during CA (Table S6), indicating that ATP can act as a crucial signalling molecule in energy homeostasis during CA^27^.

The accumulation of amino acids is a common occurrence in many plant species when subjected to cold stress^28–30^, reflecting amino acid production or related protein degradation. It is thought that many amino acids that are specifically enriched in plants have a beneficial function during stress acclimation^14^. Proline is one of the best known osmoprotectants and molecular chaperones in plants, as it plays active roles in quenching ROS, stabilizing the structure of proteins and maintaining cellular homeostasis^31,32^. Similar to that which occurred in *Thellungiella halophyla* under cold conditions^33^, proline may be involved in the defence against subsequent cold stress when the level of this amino acid is relatively high. An increase in the abundance of the aromatic amino acids phenylalanine and tyrosine, which are the precursors of the shikimate-phenylpropanoid pathway, was also induced by CA. It has been reported that low temperature can increase the accumulation of phenylalanine^34^, and exogenous application of phenylalanine can promote chilling tolerance in tomato by activating ROS-scavenging systems^35^. Our results showed an increased content of tyrosine, which may also act as an important component in the phenylpropanoid pathway for secondary metabolism. The abundance of branched-chain amino acids (BCAAs) valine and leucine, which can be synthesized from pyruvate via *de novo* methods, markedly increased in our study. These BCAAs serve as compatible osmolytes and function as alternative electron donors in the mitochondrial electron transport chain to produce ATP under environmental stress conditions, including cold stress^29,36^.

The TCA cycle (also known as the Krebs cycle) is a universal feature of metabolism that drives ATP synthesis. The conversion of pyruvate-derived acetyl-CoA and oxaloacetate to citrate is the first step of this process. Many intermediates from the TCA cycle have been shown to function in improving plant stress tolerance. For example, citrate and malate are two major organic acids that act as potential ligands for heavy metals^37^. Sebastian and Prasad reported that supplementation with citrate and malate could alleviate cadmium stress by reducing Cd-induced iron nutrition deficiency in rice^38^. The accumulation of citrate and malate was also detected in plants under cold conditions^29,39^. α-Ketoglutarate bridges the metabolism of carbohydrates and amino acids, serving as a precursor for the biosynthesis of glutamate and proline and subsequent protein biosynthesis. A possible role of α-ketoglutarate in cold stress tolerance is associated with the promotion of the antioxidant system capability and the synthesis of amino acids^40^. Our results showed that four important intermediates (citrate, α-ketoglutarate, succinate and malate) and the expression levels of two enzyme-coding genes (including those of SDH and ICL) were upregulated during CA (Fig. 6, Table S6). This outcome implied that the TCA cycle was activated and to provide the energy required by cold-acclimated tobacco.

Lignin is a polymer of phenylpropanoid compounds that participate in the synthesis of the cell wall^41^. Plants can alter their lignin content in response to abiotic stress, such as low temperature, water deficit and excess light^42^. Higher activities of lignin synthesis enzymes and increased expression levels of genes involved in lignin biosynthesis have been observed in many plant species after cold treatment^43–45^. Flavonoids are also an important diverse group of polyphenolic compounds and, structurally, are a kind of secondary metabolite with multitude biological functions in plants. It has been reported that flavonoids can serve as antioxidants, bind phytotoxins and regulate the transport of auxin during stress responses^46^. In addition, a previous study demonstrated that the content of major flavonols and the expression levels of genes in the phenylpropanoid and flavonoid pathways markedly increased at low temperature^18^. Similarly, the results of our study revealed that the expression levels of phenylpropanoid biosynthesis pathway-related genes and intermediate metabolites were upregulated under CA (Fig. 7, Table S6), suggesting that the activation of both the transcriptional cascade and bioactive metabolites involved in lignin and flavonoid anabolism provides crucial functions during CA.

**Figure 8:**
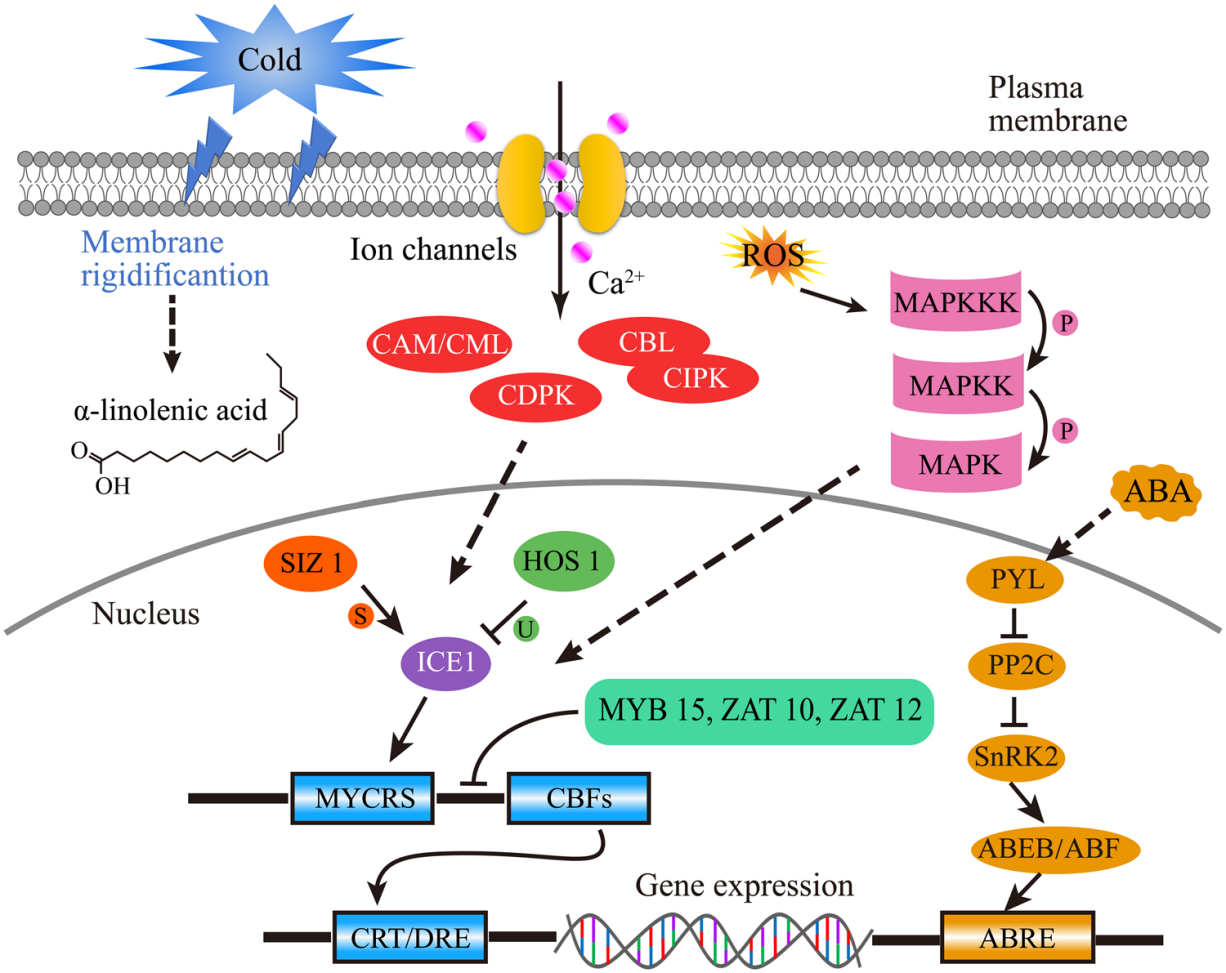
Regulation of multiple signalling pathways during cold acclimation (CA) in tobacco. Arrows indicate positive regulation, whereas lines ending with a bar indicate negative regulation. Abbreviations: CAM, calmodulin; CML, calmodulin-like; CBL, calcineurin B-like; CIPK, CBL-interacting protein kinase, CDPK, calcium-dependent protein kinase; SIZ 1, SAP and Miz 1; HOS 1, high expression of osmotically responsive gene 1; ICE1, inducer of CBF expression 1; MYCRS, MYC transcription factor recognition sequence; CBFs, C-repeat binding factors; CRT/DRE, C-repeat/dehydration-responsive elements; PYL, PYR like of ABA receptor; PP2C, protein phosphatase 2C; SnRK2, SNF1-related kinase 2; AREB/ABF, ABRE-binding protein/ABRE-binding factor; ABRE, ABA-responsive element; P, phosphorylation; U, ubiquitination; S, sumoylation.

Our GO and KEGG functional enrichment analyses revealed that “protein phosphorylation”, “protein ubiquitination”, “cellular protein modification process” and “signal transduction” were highly enriched with the DEGs, highlighting the importance of many genes encoding crucial enzymes, phosphorylation kinases and TFs during CA. Cold can lead to conformational changes in the cell membrane, which result in an increase in membrane rigidification and induce the production of secondary messengers such as Ca^2+^ molecules^47^. In our work, a large number of DEGs related to calmodulin (CAM), calmodulin-like (CML), calcineurin B-like (CBL), CBL-interacting protein kinase (CIPK) and calcium-dependent protein kinase (CDPK) were upregulated during CA treatment (Table S7). These genes are important candidates for sensing intracellular Ca^2+^ levels and for initiating calcium-directed signalling processes^48,49^ (Fig. 8). The mitogen-activated protein kinase (MAPK) cascade, which involves three protein kinases (MAPKKK-MAPKK-MAPK), functions in the cold response of plants^50^ (Fig. 8). The expression of seven genes involved in this cascade (3 MAPKKKs, 2 MAPKKs, 2 MAPKs) also increased during CA (Table S7). Moreover, the fluidity of the membrane lipids of plants can be restored by increases in unsaturated fatty acid levels when plants are subjected to cold stress^51^. The desaturation level of phospholipids noticeably increases during CA in *Arabidopsis*^52^. Similarly, the unsaturated fatty acids oleic acid and linolenic acid were upregulated under CA. This dynamic adjustment of membrane fluidity by the release of α-linolenic acid could increase the ability of plants to acclimate to abiotic stress^53^.

The ICE1-CBF-dependent cold transcriptional cascade is the best characterized regulatory pathway. Inducer of CBF expression 1 (ICE1) encodes an MYC-type basic helix-loop-helix (bHLH) TF that directly binds to the promoter of CBF/DREB, thereby activating its gene expression^11^ (Fig. 8). In our study, the expression of a homologous gene of ICE1 was upregulated (Table S7), showing a positive role in cold tolerance. Furthermore, the expression level of ICE1 is also regulated by posttranslational modifications, including ubiquitin-dependent protein degradation and small ubiquitin-related modifier (SUMO) conjugation^54^. For example, the ubiquitin E3 ligase HOS 1 (high expression of osmotically responsive gene 1) can lead to ICE1 degradation via the 26 S proteasome pathway and exerts a negative influence on the cold response^55^. Nevertheless, sumoylation by SAP and Miz 1 (SIZ 1) can enhance ICE1 stability by blocking its ubiquitination^56^. Our results showed that the expression levels of most of the E3 ubiquitin protein ligases were downregulated, while those of the two SUMOs were upregulated (Table S7), indicating that these two different modifications have opposite effects on ICE 1 stability and CBF expression. In addition, the transcription of CBF is also regulated by some TFs. MYB15, an R2R3-type TF, was found to suppress CBF expression by physically binding to the CBF promoter region^57^. ZAT 10 and ZAT 12 (C2H2 zinc finger proteins) have also been suggested to have a negative effect on the expression of CBF^58,59^. Here, multiple MYB-type TFs and zinc finger proteins exhibited different regulatory patterns in cold-acclimated tobacco (Table S7), revealing the complex regulatory network involved in the recognition of CBF promoters. In addition to the traditional ICE1-CBF signalling pathways, several ABA-responsive genes were identified to be differentially expressed under CA conditions (Fig. 8, Table S7). It has been reported that some cold-responsive genes can be activated by AREB/ABF because they possess ABRE cis elements in their promoter regions^60^. Our results showed that many genes encoding ABA-receptor *NtPYL* and ABRE-binding proteins were upregulated (Table S7). Previous evidence has reported that ABA plays an essential role in responding to low temperature^61^, suggesting that CA could motivate ABA-dependent stress signals and thus the metabolome to promote tobacco tolerance against cold stress.

## Conclusions

In present study, we comprehensively analysed the changes in the abundance of mRNA sequences and metabolites from tobacco leaves during CA. The results revealed that CA broadly activated cold signalling pathways and triggered the accumulation of a large number of stress-associated metabolites such as amino acids and sugars. The integrated analysis of the gene-metabolite network illustrated the mobilization and transport of energy from glycolysis to enhance the TCA cycle activity during CA. CA also promoted the expression of genes and the accumulation of metabolites involved in phenylpropanoids biosynthesis pathway, indicating the essential roles of these secondary metabolites under CA condition. These findings provide a comprehensive understanding of how the regulatory mechanism of genes and metabolites is reprogrammed during CA and provide valuable information for further functional characterization of the marker genes identified in this study.

## Materials and methods

### Plant materials and growth conditions

Tobacco (*Nicotiana tabacum* L.) ecotype K326 was used in this study. The seeds were first sterilized in 75% ethanol for 10 min, washed with sterile water five times and then cultivated in plastic pots in an illuminated incubator (16 h, 26 °C/8 h, 24 °C day/night with 70% relative humidity). Tobacco seedlings at the five-leaf stage were used in our work. For CA, tobacco plants were transferred to 15 °C conditions for 6 h. After 6 h of CA, the seedlings grown under normal growth conditions (CK) and those grown under CA conditions were exposed to cold stress (4 °C) for 6 h to measure the physiological changes. To detect gene expression and metabolite reprogramming during CA, the third leaves of the CA (CA_0 h and CA_6 h) treatment seedlings were harvested. Transcriptomic and metabolomic experiments were performed with three biological replicates and six biological replicates per treatment, respectively. All samples were immediately frozen in liquid nitrogen and stored at −80 °C.

### Physiological measurements of tobacco seedlings during CA and cold stress

The FW of tobacco plants at each sampling point was measured immediately after harvest, and the DW of seedlings was determined after drying at 105 °C for 1 h and then drying at 80 °C until constant weight. EL was measured according to the methods of Zhang *et al*.^62^. Briefly, the harvested leaves were immersed in ddH_2_O (20 ml) in a clean tube containing the same volume of ddH_2_O as that in the control. Both tubes were shaken at 20 rpm for 2 h before measurement of the initial conductivities of the sample (C1) and control (CK1) by a DSS-307 conductivity meter (SPSIC, China). Then, the tubes were boiled for 10 min, after which they were cooled to 25 °C, followed by the second conductivity measurement (C2 and CK2). The EL was measured as the relative conductance (C) calculated using the equation C (%) = (C1 − CK1)/(C2 − CK2) × 100. The MDA content in the tobacco leaves was measured using a kit (Nanjing Jiancheng Bioengineering Institute, Nanjing, China) according to the manufacturer’s instructions. Each treatment included five biological replicates.

### RNA isolation and transcriptomic analysis

Total RNA was isolated from tobacco leaves using TRIzol reagent (Invitrogen, Carlsbad, USA). The concentration and quality of the RNA samples were determined using a Nanodrop 2000 instrument (Thermo, Waltham, USA) and an Agilent 2100 bioanalyzer (Agilent, Palo Alto, USA). RNA-seq libraries were constructed using an Illumina TruSeq RNA Sample Prep Kit (Illumina, San Diego, USA) according to the manufacturer’s protocol and were sequenced on the Illumina HiSeq X ten platform (Illumina, San Diego, USA)^63^. After removing the adapters and low-quality sequence reads from the raw reads by Trimmomatic^64^, the clean reads were mapped to the tobacco genome (ftp://ftp.solgenomics.net/genomes/Nicotiana_tabacum/) using HISAT2^65^. The gene expression levels in all six libraries were expressed as fragments per kilobase of the transcripts per million fragments mapped (FPKM). DEGs were detected by edgeR^66^ with a false discovery rate-adjusted *p* value (FDR) < 0.05 and fold change >4. GO enrichment analysis of the DEGs was conducted using the GOseq R package on the basis of the Wallenius noncentral hypergeometric distribution; when the FDR < 0.05, the function is recognized as an enrichment item^67^. The DEGs in KEGG pathways were determined by KOBAS software based on the hypergeometric Benjamini and Hochberg hypergeometric method with default settings^68,69^ and annotated with the Automatic Annotation Server using default settings against *Arabidopsis* species^70,71^. The value of FDR < 0.05 was the threshold for DEGs enrichment in the KEGG pathways. The permission to publish the KEGG pathway map image of phenylpropanoid biosynthesis was granted by Kanehisa Laboratories.

### LC-MS/MS metabolite measurements and data analysis

The metabolites in tobacco samples were measured according to the protocol of the laboratory (Novogene Bioinformatics Technology Co., Ltd.). Briefly, freeze-dried tobacco leaves were ground to a powder and resuspended in extraction solvent containing 80% methanol. The mixtures were then incubated for 1 h and centrifuged at 14000 × g for 20 min at 4 °C. The supernatants were subsequently dried in a vacuum concentrator and reconstituted with 60% methanol for further analysis. LC-MS/MS analyses were performed on a Vanquish UHPLC system (Thermo, Waltham, USA) coupled with an Orbitrap Q Exactive HF-X mass spectrometer (Thermo, Waltham, USA). A Hypersil Gold column (100 × 2.1 mm, 1.9 μm, Thermo, Waltham, USA) was used. The samples were analysed in both positive and negative ion modes. The solvent gradient was set as follows: 2% B, 1.5 min; 2–100% B, 12 min; 100% B, 14 min; 100-2% B, 14.1 min; and 2% B, 16 min. The mass spectrometer was operated in positive mode and negative mode with a spray voltage of 3200 V and capillary temperature of 320 °C. The data extracted from chromatograms were processed, calibrated and filtered by in-house software. The annotations of the detected accurate metabolites were queried and compared to a laboratory database (Novogene Bioinformatics Technology Co., Ltd.). SIMCA-P (version 14.1, Umetrics, Umea, Sweden) was used to perform multivariate statistical analysis^72^. After Pareto scaling, PCA and orthogonal partial least squares discrimination analysis (OPLS-DA) were performed for unsupervised multivariate statistical analysis and supervised analysis, respectively, to identify important variables with discriminative power. VIP is the weighted sum of the squares of the OPLS-DA analysis, which indicates the importance of a variable to the entire model. The significantly different metabolites were determined based on the combination of a statistically significant threshold of a VIP > 1.0, a Student’s t-test *p* < 0.05 and FC > 1.5 or <0.66.

### RT-qPCR analysis

The same RNA samples for the transcriptomic analysis were used in the RT-qPCR experiment. cDNA was generated using a PrimeScript cDNA Synthesis Kit (Takara, Dalian, China), and RT-qPCR with SYBR Green Supermix was performed on an ABI StepOne instrument (Takara, Dalian, China) according to the manufacturer’s instructions. The tobacco *ef1α* gene was used as an internal control. The relative expression levels of select genes were measured for three independent biological replicates and were calculated using the 2^−ΔΔCT^ method^73^.

### Statistical analysis

The significant differences in the FW, DW, EL and MDA content of the tobacco seedlings between the CK and cold-acclimated tobacco plants at each sampling time were evaluated by one-way ANOVA followed by LSD post hoc tests, performed by SPSS (19.0, IBM Corp, Armonk, USA).

## Supplementary information

Supplementary Figure S1

Supplementary Table S1

Supplementary Table S2

Supplementary Table S3

Supplementary Table S4

Supplementary Table S5

Supplementary Table S6

Supplementary Table S7

## Data Availability

The transcriptomic data were uploaded to the sequence read archive (SRA) at NCBI (Accession Number PRJNA600629). All other additional data sets supporting the results of this article are included in this article and its supplementary information files.
