# Supplementary material for: Combined transcriptomic and metabolomic analyses uncover rearranged gene expression and metabolite metabolism in tobacco during cold acclimation
Source: Sci Rep. 2020 Mar 23;10:5242. doi: 10.1038/s41598-020-62111-x (PMC7090041; doi:10.1038/s41598-020-62111-x)
Supplement: Supplementary file 1 — Supplementary Figure S1 [file 41598_2020_62111_MOESM1_ESM.docx]

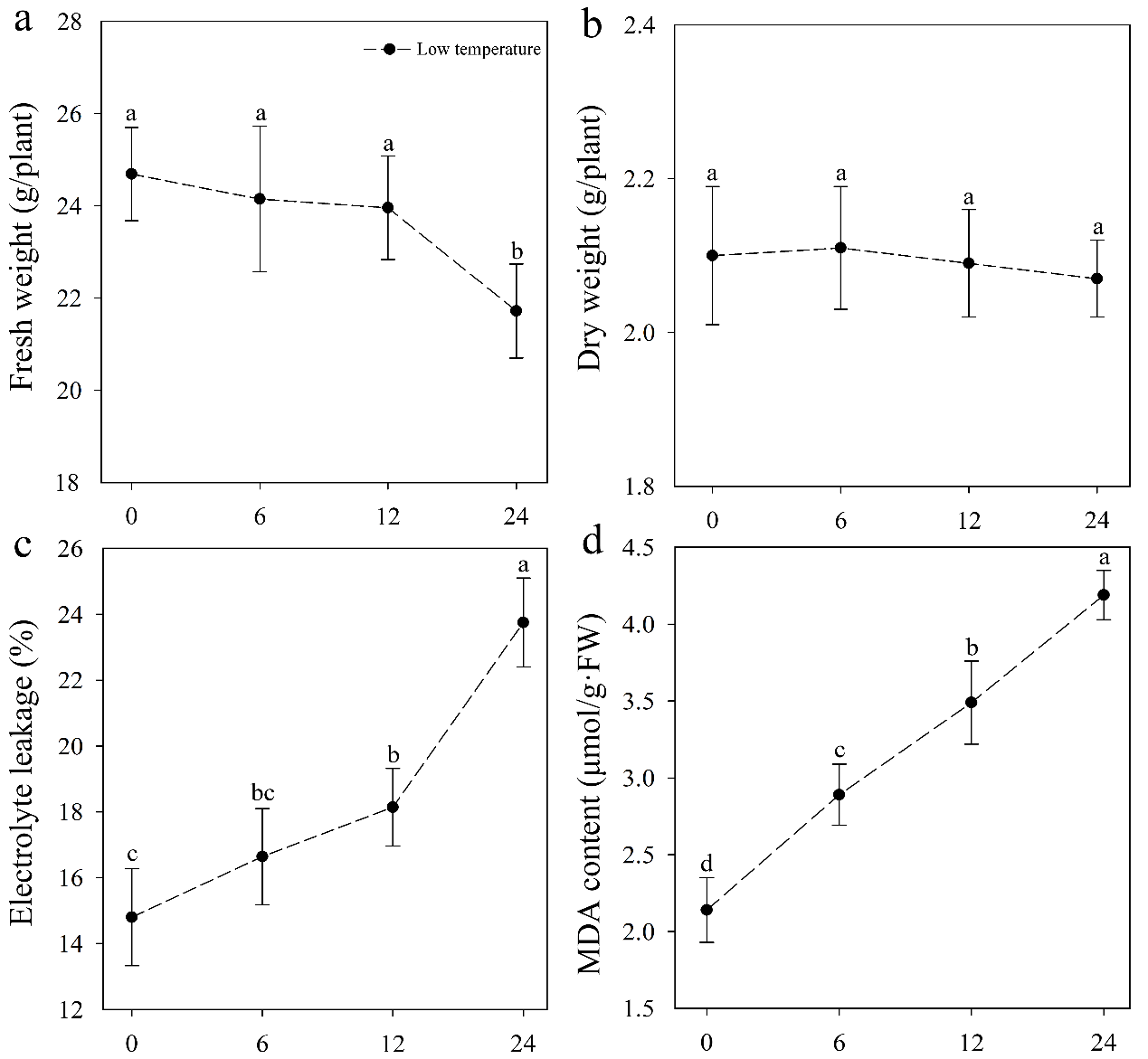


Fig. S1 Physiological changes in tobacco plants under low temperature. Effect of low temperature on the fresh weight (a), dry weight (b), electrolyte leakage (c) and MDA content (d) of tobacco seedlings. Values are the means ± SDs (n = 5). Different letters indicate a significant difference at *p* < 0.05 evaluated by the LSD test.
